# [*O*-Ethyl (*Z*)-*N*-(2-chloro­phen­yl)thio­carbamato-κ*S*](tricyclo­hexyl­phosphine-κ*P*)gold(I)

**DOI:** 10.1107/S160053681001189X

**Published:** 2010-04-10

**Authors:** Primjira P. Tadbuppa, Edward R. T. Tiekink

**Affiliations:** a Department of Chemistry, National University of Singapore, Singapore 117543; b Department of Chemistry, University of Malaya, 50603 Kuala Lumpur, Malaysia

## Abstract

The title compound, [Au(C_9_H_9_ClNOS)(C_18_H_33_P)], features a slightly distorted linear coordination geometry for the Au atom defined by a *S*,*P*-donor set [S—Au—P = 177.62 (5)°]. The distortion is ascribed to the close approach of the O atom, which forms an intra­molecular contact of 2.970 (5) Å. Disorder was found in the structure with two positions of equal weight being resolved for the C atoms comprising the eth­oxy group.

## Related literature

For the structural systematics and luminescence properties of phosphinegold(I) carbonimidothio­ates, see: Ho *et al.* (2006 ▶); Ho & Tiekink (2007 ▶); Kuan *et al.* (2008 ▶). For the synthesis, see: Hall *et al.* (1993 ▶).
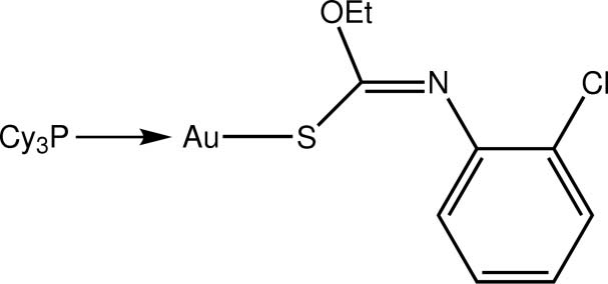


## Experimental

### 

#### Crystal data

[Au(C_9_H_9_ClNOS)(C_18_H_33_P)]
*M* *_r_* = 692.06
Monoclinic, 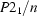

*a* = 12.0513 (11) Å
*b* = 18.2460 (16) Å
*c* = 13.9712 (12) Å
β = 108.892 (2)°
*V* = 2906.6 (4) Å^3^
*Z* = 4
Mo *K*α radiation
μ = 5.30 mm^−1^
*T* = 223 K
0.16 × 0.13 × 0.08 mm

#### Data collection

Bruker SMART CCD diffractometer
Absorption correction: multi-scan (*SADABS*; Bruker, 2000 ▶) *T*_min_ = 0.294, *T*_max_ = 1.000
20508 measured reflections
6663 independent reflections
5016 reflections with *I* > 2σ(*I*)
*R*_int_ = 0.045

#### Refinement

*R*[*F* ^2^ > 2σ(*F* ^2^)] = 0.037
*wR*(*F* ^2^) = 0.113
*S* = 1.07
6663 reflections
307 parameters
4 restraints
H-atom parameters constrained
Δρ_max_ = 1.09 e Å^−3^
Δρ_min_ = −0.76 e Å^−3^

### 

Data collection: *SMART* (Bruker, 2000 ▶); cell refinement: *SAINT* (Bruker, 2000 ▶); data reduction: *SAINT*; program(s) used to solve structure: *PATTY* in *DIRDIF92* (Beurskens *et al.*, 1992 ▶); program(s) used to refine structure: *SHELXL97* (Sheldrick, 2008 ▶); molecular graphics: *ORTEP-3* (Farrugia, 1997 ▶) and *DIAMOND* (Brandenburg, 2006 ▶); software used to prepare material for publication: *publCIF* (Westrip, 2010 ▶).

## Supplementary Material

Crystal structure: contains datablocks global, I. DOI: 10.1107/S160053681001189X/ez2205sup1.cif

Structure factors: contains datablocks I. DOI: 10.1107/S160053681001189X/ez2205Isup2.hkl

Additional supplementary materials:  crystallographic information; 3D view; checkCIF report

## Figures and Tables

**Table 1:** Selected bond lengths (Å)

Au—P1	2.2648 (13)
Au—S1	2.3060 (14)

**Fig. 1.**
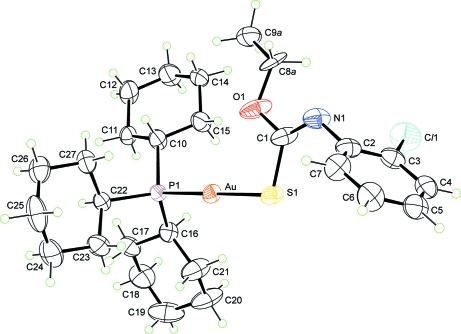
Molecular structure of (I) showing atom-labelling scheme and displacement ellipsoids at the 35% probability level.

## supplementary crystallographic information

### Comment

The synthesis and characterisation of the title compound, (I), was investigated in the context of crystal engineering and luminescence studies of molecules of the type R_3_PAu[SC(OR')═NR''], for R, R' and R'' = alkyl and aryl (Ho *et al.* 2006; Ho & Tiekink, 2007; Kuan *et al.*, 2008).

The Au atom in (I), Fig. 1, is linearly coordinated within a *SP* donor set, Table 1. The thiocarbamate ligand coordinates as a thiolate as seen in the C1–S1 [1.734 (6) Å] and C1═N1 [1.261 (8) Å] distances. A small deviation from the ideal geometry is noted, Table 1, which is ascribed to the close approach of the O1 atom [2.970 (5) Å]. No specific intermolecular interactions are noted in the crystal packing.

### Experimental

Compound (I) was prepared following the standard literature procedure from the reaction of Cy_3_PAuCl and EtOC(═S)N(H)(C_6_H_4_Cl-2) in the presence of NaOH (Hall *et al.*, 1993). Crystals were obtained by the slow evaporation of a CH_2_Cl_2_/hexane (3/1) solution held at room temperature.

### Refinement

The H atoms were geometrically placed (C—H = 0.94-0.99 Å) and refined as riding with *U*_iso_(H) = 1.2-1.5*U*_eq_(C). The atoms comprising the ethoxy group were found to be disordered with two positions resolved for the C8 and C9 atoms. From anisotropic refinement, the site occupancy factors were found to be experimentally equivalent and therefore, fixed at 0.5 in the final cycles of the refinement. The anisotropic displacement parameters for each of the pairs of C8 and C9 atoms were constrained to be equivalent. The maximum and minimum residual electron density peaks of 1.09 and 0.76 e Å^-3^, respectively, were located 0.82 Å and 0.86 Å from the Au atom.

### Crystal data

**Table:**

[Au(C_9_H_9_ClNOS)(C_18_H_33_P)]	*F*(000) = 1384
*M**_r_* = 692.06	*D*_x_ = 1.581 Mg m^−^^3^
Monoclinic, *P*2_1_/*n*	Mo *K*α radiation, λ = 0.71069 Å
Hall symbol: -P 2yn	Cell parameters from 4436 reflections
*a* = 12.0513 (11) Å	θ = 2.1–25.0°
*b* = 18.2460 (16) Å	µ = 5.30 mm^−^^1^
*c* = 13.9712 (12) Å	*T* = 223 K
β = 108.892 (2)°	Block, colourless
*V* = 2906.6 (4) Å^3^	0.16 × 0.13 × 0.08 mm
*Z* = 4	

### Data collection

**Table:**

Bruker SMART CCD diffractometer	6663 independent reflections
Radiation source: fine-focus sealed tube	5016 reflections with *I* > 2σ(*I*)
graphite	*R*_int_ = 0.045
ω scans	θ_max_ = 27.5°, θ_min_ = 1.9°
Absorption correction: multi-scan (*SADABS*; Bruker, 2000)	*h* = −15→14
*T*_min_ = 0.294, *T*_max_ = 1.000	*k* = −22→23
20508 measured reflections	*l* = −18→18

### Refinement

**Table:**

Refinement on *F*^2^	Primary atom site location: structure-invariant direct methods
Least-squares matrix: full	Secondary atom site location: difference Fourier map
*R*[*F*^2^ > 2σ(*F*^2^)] = 0.037	Hydrogen site location: inferred from neighbouring sites
*wR*(*F*^2^) = 0.113	H-atom parameters constrained
*S* = 1.07	*w* = 1/[σ^2^(*F*_o_^2^) + (0.0581*P*)^2^] where *P* = (*F*_o_^2^ + 2*F*_c_^2^)/3
6663 reflections	(Δ/σ)_max_ = 0.002
307 parameters	Δρ_max_ = 1.09 e Å^−^^3^
4 restraints	Δρ_min_ = −0.76 e Å^−^^3^

### Special details

**Table:**

Geometry. All s.u.'s (except the s.u. in the dihedral angle between two l.s. planes) are estimated using the full covariance matrix. The cell s.u.'s are taken into account individually in the estimation of s.u.'s in distances, angles and torsion angles; correlations between s.u.'s in cell parameters are only used when they are defined by crystal symmetry. An approximate (isotropic) treatment of cell s.u.'s is used for estimating s.u.'s involving l.s. planes.
Refinement. Refinement of *F*^2^ against ALL reflections. The weighted *R*-factor wR and goodness of fit *S* are based on *F*^2^, conventional *R*-factors *R* are based on *F*, with *F* set to zero for negative *F*^2^. The threshold expression of *F*^2^ > 2σ(*F*^2^) is used only for calculating *R*-factors(gt) etc. and is not relevant to the choice of reflections for refinement. *R*-factors based on *F*^2^ are statistically about twice as large as those based on *F*, and *R*- factors based on ALL data will be even larger.

### Fractional atomic coordinates and isotropic or equivalent isotropic displacement parameters (Å^2^)

**Table:**

	*x*	*y*	*z*	*U*_iso_*/*U*_eq_	Occ. (<1)
Au	0.529378 (18)	0.173673 (11)	0.875172 (14)	0.04429 (9)	
Cl1	0.42395 (18)	0.22916 (13)	1.26094 (19)	0.1023 (7)	
S1	0.59973 (15)	0.18353 (8)	1.04903 (11)	0.0560 (4)	
P1	0.45520 (12)	0.16794 (7)	0.70427 (10)	0.0395 (3)	
N1	0.5235 (5)	0.1057 (3)	1.1784 (4)	0.0663 (15)	
C1	0.5164 (6)	0.1201 (3)	1.0884 (5)	0.0610 (16)	
C2	0.6046 (6)	0.1433 (3)	1.2587 (4)	0.0581 (15)	
C3	0.5696 (6)	0.2006 (4)	1.3065 (5)	0.0594 (15)	
C4	0.6477 (7)	0.2369 (4)	1.3876 (5)	0.0692 (18)	
H4	0.6220	0.2760	1.4190	0.083*	
C5	0.7621 (7)	0.2153 (4)	1.4216 (5)	0.0707 (19)	
H5	0.8159	0.2400	1.4762	0.085*	
C6	0.7986 (7)	0.1581 (4)	1.3767 (5)	0.073 (2)	
H6	0.8773	0.1428	1.4009	0.087*	
C7	0.7210 (6)	0.1229 (4)	1.2964 (5)	0.0693 (17)	
H7	0.7476	0.0837	1.2660	0.083*	
O1A	0.4364 (5)	0.0846 (2)	1.0101 (3)	0.0862 (17)	0.50
C8A	0.3522 (11)	0.0437 (7)	1.0452 (10)	0.070 (4)	0.50
H8A1	0.3905	0.0004	1.0831	0.083*	0.50
H8A2	0.3253	0.0746	1.0907	0.083*	0.50
C9A	0.2477 (12)	0.0201 (8)	0.9560 (10)	0.078 (3)	0.50
H9A1	0.1995	0.0625	0.9281	0.116*	0.50
H9A2	0.2017	−0.0155	0.9785	0.116*	0.50
H9A3	0.2752	−0.0017	0.9045	0.116*	0.50
O1B	0.4364 (5)	0.0846 (2)	1.0101 (3)	0.0862 (17)	0.50
C8B	0.3756 (12)	0.0190 (5)	1.0270 (13)	0.070 (4)	0.50
H8B1	0.4211	−0.0080	1.0876	0.083*	0.50
H8B2	0.3530	−0.0137	0.9682	0.083*	0.50
C9B	0.2715 (13)	0.0587 (8)	1.0410 (12)	0.078 (3)	0.50
H9B1	0.2951	0.0823	1.1068	0.116*	0.50
H9B2	0.2094	0.0238	1.0367	0.116*	0.50
H9B3	0.2434	0.0955	0.9886	0.116*	0.50
C10	0.3110 (5)	0.1225 (4)	0.6683 (4)	0.0550 (14)	
H10	0.3306	0.0707	0.6873	0.066*	
C11	0.2437 (5)	0.1186 (3)	0.5555 (4)	0.0473 (12)	
H11A	0.2281	0.1685	0.5285	0.057*	
H11B	0.2924	0.0941	0.5210	0.057*	
C12	0.1291 (6)	0.0781 (5)	0.5329 (5)	0.079 (2)	
H12A	0.0834	0.0864	0.4617	0.094*	
H12B	0.1461	0.0255	0.5413	0.094*	
C13	0.0565 (6)	0.0979 (5)	0.5947 (5)	0.083 (2)	
H13A	−0.0070	0.0621	0.5834	0.100*	
H13B	0.0208	0.1460	0.5730	0.100*	
C14	0.1252 (6)	0.1008 (4)	0.7069 (5)	0.0602 (16)	
H14A	0.0757	0.1227	0.7428	0.072*	
H14B	0.1441	0.0507	0.7321	0.072*	
C15	0.2376 (6)	0.1444 (4)	0.7308 (5)	0.0644 (16)	
H15A	0.2183	0.1966	0.7198	0.077*	
H15B	0.2830	0.1378	0.8024	0.077*	
C16	0.4327 (6)	0.2590 (3)	0.6469 (4)	0.0536 (14)	
H16	0.3527	0.2735	0.6447	0.064*	
C17	0.4317 (6)	0.2634 (4)	0.5363 (5)	0.0648 (17)	
H17A	0.5074	0.2464	0.5328	0.078*	
H17B	0.3707	0.2308	0.4943	0.078*	
C18	0.4091 (9)	0.3410 (4)	0.4943 (7)	0.088 (3)	
H18A	0.4169	0.3421	0.4266	0.106*	
H18B	0.3287	0.3553	0.4880	0.106*	
C19	0.4925 (10)	0.3944 (5)	0.5605 (9)	0.125 (4)	
H19A	0.4737	0.4438	0.5324	0.150*	
H19B	0.5724	0.3826	0.5620	0.150*	
C20	0.4871 (10)	0.3932 (4)	0.6706 (8)	0.111 (3)	
H20A	0.5444	0.4278	0.7129	0.134*	
H20B	0.4088	0.4082	0.6702	0.134*	
C21	0.5138 (9)	0.3155 (4)	0.7145 (7)	0.096 (3)	
H21A	0.5953	0.3030	0.7222	0.115*	
H21B	0.5048	0.3142	0.7818	0.115*	
C22	0.5459 (5)	0.1134 (3)	0.6470 (4)	0.0447 (12)	
H22	0.5119	0.1177	0.5725	0.054*	
C23	0.6710 (6)	0.1432 (4)	0.6789 (6)	0.0699 (17)	
H23A	0.6698	0.1934	0.6535	0.084*	
H23B	0.7028	0.1449	0.7529	0.084*	
C24	0.7504 (7)	0.0962 (5)	0.6385 (6)	0.086 (2)	
H24A	0.7234	0.0985	0.5644	0.104*	
H24B	0.8306	0.1155	0.6632	0.104*	
C25	0.7504 (7)	0.0176 (5)	0.6717 (5)	0.090 (3)	
H25A	0.8002	−0.0118	0.6433	0.109*	
H25B	0.7828	0.0148	0.7456	0.109*	
C26	0.6284 (7)	−0.0126 (4)	0.6372 (5)	0.0731 (19)	
H26A	0.6300	−0.0631	0.6616	0.088*	
H26B	0.5992	−0.0138	0.5631	0.088*	
C27	0.5449 (6)	0.0325 (3)	0.6751 (5)	0.0597 (16)	
H27A	0.5676	0.0279	0.7489	0.072*	
H27B	0.4652	0.0131	0.6460	0.072*	

### Atomic displacement parameters (Å^2^)

**Table:**

	*U*^11^	*U*^22^	*U*^33^	*U*^12^	*U*^13^	*U*^23^
Au	0.04234 (14)	0.04968 (14)	0.03387 (12)	−0.00306 (9)	0.00269 (9)	−0.00253 (8)
Cl1	0.0602 (12)	0.1215 (18)	0.1212 (18)	0.0090 (11)	0.0236 (12)	0.0049 (14)
S1	0.0604 (9)	0.0607 (9)	0.0355 (7)	−0.0221 (7)	−0.0003 (6)	−0.0001 (6)
P1	0.0340 (7)	0.0486 (7)	0.0338 (6)	−0.0022 (5)	0.0080 (5)	−0.0011 (5)
N1	0.070 (4)	0.070 (3)	0.046 (3)	−0.019 (3)	0.001 (3)	0.018 (2)
C1	0.064 (4)	0.048 (3)	0.053 (3)	−0.018 (3)	−0.007 (3)	0.012 (3)
C2	0.058 (4)	0.063 (4)	0.046 (3)	−0.013 (3)	0.007 (3)	0.017 (3)
C3	0.055 (4)	0.070 (4)	0.053 (3)	−0.002 (3)	0.018 (3)	0.020 (3)
C4	0.079 (5)	0.081 (5)	0.053 (4)	−0.016 (4)	0.030 (4)	−0.001 (3)
C5	0.073 (5)	0.095 (5)	0.038 (3)	−0.018 (4)	0.008 (3)	0.005 (3)
C6	0.060 (4)	0.098 (5)	0.048 (4)	0.008 (4)	−0.001 (3)	0.011 (3)
C7	0.065 (4)	0.073 (4)	0.058 (4)	0.008 (3)	0.005 (3)	0.008 (3)
O1A	0.101 (4)	0.070 (3)	0.057 (3)	−0.044 (3)	−0.019 (3)	0.014 (2)
C8A	0.094 (8)	0.018 (6)	0.074 (7)	−0.022 (6)	−0.003 (5)	−0.001 (6)
C9A	0.076 (7)	0.080 (7)	0.079 (7)	−0.030 (6)	0.027 (6)	−0.009 (5)
O1B	0.101 (4)	0.070 (3)	0.057 (3)	−0.044 (3)	−0.019 (3)	0.014 (2)
C8B	0.094 (8)	0.018 (6)	0.074 (7)	−0.022 (6)	−0.003 (5)	−0.001 (6)
C9B	0.076 (7)	0.080 (7)	0.079 (7)	−0.030 (6)	0.027 (6)	−0.009 (5)
C10	0.041 (3)	0.082 (4)	0.040 (3)	−0.010 (3)	0.010 (2)	−0.012 (3)
C11	0.043 (3)	0.057 (3)	0.038 (3)	−0.001 (2)	0.008 (2)	−0.007 (2)
C12	0.050 (4)	0.115 (6)	0.067 (4)	−0.016 (4)	0.013 (3)	−0.039 (4)
C13	0.058 (4)	0.119 (6)	0.070 (5)	−0.028 (4)	0.017 (4)	−0.022 (4)
C14	0.054 (4)	0.071 (4)	0.063 (4)	−0.015 (3)	0.029 (3)	−0.011 (3)
C15	0.057 (4)	0.091 (4)	0.045 (3)	−0.018 (4)	0.016 (3)	−0.008 (3)
C16	0.057 (4)	0.056 (3)	0.045 (3)	0.004 (3)	0.013 (3)	0.004 (3)
C17	0.073 (5)	0.070 (4)	0.057 (4)	0.000 (3)	0.028 (3)	0.018 (3)
C18	0.106 (7)	0.077 (5)	0.086 (6)	0.017 (5)	0.035 (5)	0.038 (4)
C19	0.123 (9)	0.087 (6)	0.162 (10)	−0.022 (6)	0.043 (8)	0.063 (6)
C20	0.136 (9)	0.052 (4)	0.126 (8)	−0.035 (5)	0.015 (7)	0.009 (5)
C21	0.116 (8)	0.065 (5)	0.090 (6)	−0.027 (4)	0.009 (6)	0.005 (4)
C22	0.040 (3)	0.055 (3)	0.041 (3)	0.002 (2)	0.014 (2)	0.000 (2)
C23	0.049 (4)	0.079 (4)	0.087 (5)	−0.002 (3)	0.029 (4)	−0.003 (4)
C24	0.055 (4)	0.112 (6)	0.097 (6)	0.022 (4)	0.033 (4)	−0.001 (5)
C25	0.079 (5)	0.133 (7)	0.060 (4)	0.061 (5)	0.022 (4)	0.007 (4)
C26	0.085 (5)	0.063 (4)	0.074 (4)	0.026 (4)	0.029 (4)	0.006 (3)
C27	0.071 (4)	0.058 (4)	0.055 (3)	0.016 (3)	0.028 (3)	0.009 (3)

### Geometric parameters (Å, °)

**Table:**

Au—P1	2.2648 (13)	C12—H12B	0.9800
Au—S1	2.3060 (14)	C13—C14	1.518 (9)
Cl1—C3	1.742 (7)	C13—H13A	0.9800
S1—C1	1.734 (6)	C13—H13B	0.9800
P1—C16	1.826 (6)	C14—C15	1.512 (8)
P1—C10	1.842 (6)	C14—H14A	0.9800
P1—C22	1.842 (5)	C14—H14B	0.9800
N1—C1	1.261 (8)	C15—H15A	0.9800
N1—C2	1.405 (8)	C15—H15B	0.9800
C1—O1B	1.364 (7)	C16—C21	1.520 (9)
C1—O1A	1.364 (7)	C16—C17	1.544 (8)
C2—C3	1.378 (10)	C16—H16	0.9900
C2—C7	1.380 (9)	C17—C18	1.523 (9)
C3—C4	1.384 (9)	C17—H17A	0.9800
C4—C5	1.363 (10)	C17—H17B	0.9800
C4—H4	0.9400	C18—C19	1.488 (13)
C5—C6	1.363 (10)	C18—H18A	0.9800
C5—H5	0.9400	C18—H18B	0.9800
C6—C7	1.366 (9)	C19—C20	1.561 (16)
C6—H6	0.9400	C19—H19A	0.9800
C7—H7	0.9400	C19—H19B	0.9800
O1A—C8A	1.466 (5)	C20—C21	1.537 (10)
C8A—C9A	1.519 (5)	C20—H20A	0.9800
C8A—H8A1	0.9800	C20—H20B	0.9800
C8A—H8A2	0.9800	C21—H21A	0.9800
C9A—H9A1	0.9700	C21—H21B	0.9800
C9A—H9A2	0.9700	C22—C23	1.528 (8)
C9A—H9A3	0.9700	C22—C27	1.529 (7)
O1B—C8B	1.461 (5)	C22—H22	0.9900
C8B—C9B	1.515 (5)	C23—C24	1.524 (9)
C8B—H8B1	0.9800	C23—H23A	0.9800
C8B—H8B2	0.9800	C23—H23B	0.9800
C9B—H9B1	0.9700	C24—C25	1.507 (10)
C9B—H9B2	0.9700	C24—H24A	0.9800
C9B—H9B3	0.9700	C24—H24B	0.9800
C10—C15	1.485 (8)	C25—C26	1.496 (11)
C10—C11	1.522 (7)	C25—H25A	0.9800
C10—H10	0.9900	C25—H25B	0.9800
C11—C12	1.507 (8)	C26—C27	1.521 (8)
C11—H11A	0.9800	C26—H26A	0.9800
C11—H11B	0.9800	C26—H26B	0.9800
C12—C13	1.460 (10)	C27—H27A	0.9800
C12—H12A	0.9800	C27—H27B	0.9800
			
P1—Au—S1	177.62 (5)	C13—C14—H14B	108.9
C1—S1—Au	103.2 (2)	H14A—C14—H14B	107.7
C16—P1—C10	107.2 (3)	C10—C15—C14	113.0 (5)
C16—P1—C22	109.0 (3)	C10—C15—H15A	109.0
C10—P1—C22	106.1 (3)	C14—C15—H15A	109.0
C16—P1—Au	111.85 (18)	C10—C15—H15B	109.0
C10—P1—Au	108.91 (18)	C14—C15—H15B	109.0
C22—P1—Au	113.32 (18)	H15A—C15—H15B	107.8
C1—N1—C2	119.9 (5)	C21—C16—C17	112.4 (6)
N1—C1—O1B	120.2 (5)	C21—C16—P1	111.7 (4)
N1—C1—O1A	120.2 (5)	C17—C16—P1	116.0 (4)
O1B—C1—O1A	0.0 (4)	C21—C16—H16	105.2
N1—C1—S1	126.6 (5)	C17—C16—H16	105.2
O1B—C1—S1	113.2 (4)	P1—C16—H16	105.2
O1A—C1—S1	113.2 (4)	C18—C17—C16	112.1 (6)
C3—C2—C7	116.9 (6)	C18—C17—H17A	109.2
C3—C2—N1	121.0 (6)	C16—C17—H17A	109.2
C7—C2—N1	122.1 (7)	C18—C17—H17B	109.2
C2—C3—C4	121.7 (6)	C16—C17—H17B	109.2
C2—C3—Cl1	118.7 (5)	H17A—C17—H17B	107.9
C4—C3—Cl1	119.6 (6)	C19—C18—C17	111.6 (7)
C5—C4—C3	119.3 (7)	C19—C18—H18A	109.3
C5—C4—H4	120.3	C17—C18—H18A	109.3
C3—C4—H4	120.3	C19—C18—H18B	109.3
C6—C5—C4	120.1 (6)	C17—C18—H18B	109.3
C6—C5—H5	119.9	H18A—C18—H18B	108.0
C4—C5—H5	119.9	C18—C19—C20	111.6 (7)
C5—C6—C7	120.1 (7)	C18—C19—H19A	109.3
C5—C6—H6	120.0	C20—C19—H19A	109.3
C7—C6—H6	120.0	C18—C19—H19B	109.3
C6—C7—C2	121.9 (7)	C20—C19—H19B	109.3
C6—C7—H7	119.1	H19A—C19—H19B	108.0
C2—C7—H7	119.1	C21—C20—C19	109.6 (8)
C1—O1A—C8A	111.1 (6)	C21—C20—H20A	109.7
C9A—C8A—O1A	110.5 (9)	C19—C20—H20A	109.7
C9A—C8A—H8A1	109.6	C21—C20—H20B	109.7
O1A—C8A—H8A1	109.6	C19—C20—H20B	109.7
C9A—C8A—H8A2	109.6	H20A—C20—H20B	108.2
O1A—C8A—H8A2	109.6	C16—C21—C20	111.6 (7)
H8A1—C8A—H8A2	108.1	C16—C21—H21A	109.3
C1—O1B—C8B	121.3 (8)	C20—C21—H21A	109.3
O1B—C8B—C9B	96.3 (9)	C16—C21—H21B	109.3
O1B—C8B—H8B1	112.5	C20—C21—H21B	109.3
C9B—C8B—H8B1	112.5	H21A—C21—H21B	108.0
O1B—C8B—H8B2	112.5	C23—C22—C27	111.0 (5)
C9B—C8B—H8B2	112.5	C23—C22—P1	110.4 (4)
H8B1—C8B—H8B2	110.0	C27—C22—P1	110.8 (4)
C8B—C9B—H9B1	109.5	C23—C22—H22	108.2
C8B—C9B—H9B2	109.5	C27—C22—H22	108.2
H9B1—C9B—H9B2	109.5	P1—C22—H22	108.2
C8B—C9B—H9B3	109.5	C24—C23—C22	111.7 (6)
H9B1—C9B—H9B3	109.5	C24—C23—H23A	109.3
H9B2—C9B—H9B3	109.5	C22—C23—H23A	109.3
C15—C10—C11	113.7 (5)	C24—C23—H23B	109.3
C15—C10—P1	114.0 (4)	C22—C23—H23B	109.3
C11—C10—P1	116.3 (4)	H23A—C23—H23B	107.9
C15—C10—H10	103.6	C25—C24—C23	111.0 (6)
C11—C10—H10	103.6	C25—C24—H24A	109.4
P1—C10—H10	103.6	C23—C24—H24A	109.4
C12—C11—C10	112.6 (5)	C25—C24—H24B	109.4
C12—C11—H11A	109.1	C23—C24—H24B	109.4
C10—C11—H11A	109.1	H24A—C24—H24B	108.0
C12—C11—H11B	109.1	C26—C25—C24	110.5 (6)
C10—C11—H11B	109.1	C26—C25—H25A	109.6
H11A—C11—H11B	107.8	C24—C25—H25A	109.6
C13—C12—C11	116.0 (5)	C26—C25—H25B	109.6
C13—C12—H12A	108.3	C24—C25—H25B	109.6
C11—C12—H12A	108.3	H25A—C25—H25B	108.1
C13—C12—H12B	108.3	C25—C26—C27	112.3 (6)
C11—C12—H12B	108.3	C25—C26—H26A	109.1
H12A—C12—H12B	107.4	C27—C26—H26A	109.1
C12—C13—C14	112.9 (6)	C25—C26—H26B	109.1
C12—C13—H13A	109.0	C27—C26—H26B	109.1
C14—C13—H13A	109.0	H26A—C26—H26B	107.9
C12—C13—H13B	109.0	C26—C27—C22	111.8 (5)
C14—C13—H13B	109.0	C26—C27—H27A	109.3
H13A—C13—H13B	107.8	C22—C27—H27A	109.3
C15—C14—C13	113.4 (5)	C26—C27—H27B	109.3
C15—C14—H14A	108.9	C22—C27—H27B	109.3
C13—C14—H14A	108.9	H27A—C27—H27B	107.9
C15—C14—H14B	108.9		
			
P1—Au—S1—C1	95.0 (12)	C15—C10—C11—C12	−46.7 (8)
S1—Au—P1—C16	40.8 (12)	P1—C10—C11—C12	177.8 (5)
S1—Au—P1—C10	−77.6 (12)	C10—C11—C12—C13	46.4 (9)
S1—Au—P1—C22	164.6 (12)	C11—C12—C13—C14	−47.4 (10)
C2—N1—C1—O1B	−178.9 (6)	C12—C13—C14—C15	48.6 (9)
C2—N1—C1—O1A	−178.9 (6)	C11—C10—C15—C14	49.4 (8)
C2—N1—C1—S1	0.7 (10)	P1—C10—C15—C14	−174.1 (5)
Au—S1—C1—N1	179.9 (6)	C13—C14—C15—C10	−50.1 (8)
Au—S1—C1—O1B	−0.5 (6)	C10—P1—C16—C21	144.8 (6)
Au—S1—C1—O1A	−0.5 (6)	C22—P1—C16—C21	−100.7 (6)
C1—N1—C2—C3	100.5 (8)	Au—P1—C16—C21	25.4 (6)
C1—N1—C2—C7	−82.6 (8)	C10—P1—C16—C17	−84.6 (5)
C7—C2—C3—C4	1.0 (9)	C22—P1—C16—C17	29.9 (6)
N1—C2—C3—C4	178.0 (5)	Au—P1—C16—C17	156.0 (4)
C7—C2—C3—Cl1	179.3 (5)	C21—C16—C17—C18	−51.3 (9)
N1—C2—C3—Cl1	−3.6 (8)	P1—C16—C17—C18	178.4 (6)
C2—C3—C4—C5	−0.3 (9)	C16—C17—C18—C19	53.5 (10)
Cl1—C3—C4—C5	−178.6 (5)	C17—C18—C19—C20	−57.1 (11)
C3—C4—C5—C6	−0.7 (10)	C18—C19—C20—C21	57.7 (12)
C4—C5—C6—C7	1.0 (11)	C17—C16—C21—C20	52.8 (10)
C5—C6—C7—C2	−0.3 (11)	P1—C16—C21—C20	−174.8 (7)
C3—C2—C7—C6	−0.7 (10)	C19—C20—C21—C16	−55.0 (12)
N1—C2—C7—C6	−177.7 (6)	C16—P1—C22—C23	69.0 (5)
N1—C1—O1A—C8A	11.7 (11)	C10—P1—C22—C23	−175.8 (4)
O1B—C1—O1A—C8A	0(39)	Au—P1—C22—C23	−56.3 (5)
S1—C1—O1A—C8A	−167.9 (7)	C16—P1—C22—C27	−167.6 (4)
C1—O1A—C8A—C9A	166.2 (10)	C10—P1—C22—C27	−52.4 (5)
N1—C1—O1B—C8B	−13.5 (12)	Au—P1—C22—C27	67.1 (4)
O1A—C1—O1B—C8B	0(47)	C27—C22—C23—C24	52.9 (7)
S1—C1—O1B—C8B	166.9 (7)	P1—C22—C23—C24	176.2 (5)
C1—O1B—C8B—C9B	90.8 (12)	C22—C23—C24—C25	−56.3 (9)
C16—P1—C10—C15	−80.8 (5)	C23—C24—C25—C26	57.6 (8)
C22—P1—C10—C15	162.8 (5)	C24—C25—C26—C27	−56.9 (8)
Au—P1—C10—C15	40.4 (5)	C25—C26—C27—C22	54.2 (8)
C16—P1—C10—C11	54.5 (5)	C23—C22—C27—C26	−51.4 (7)
C22—P1—C10—C11	−61.9 (5)	P1—C22—C27—C26	−174.5 (5)
Au—P1—C10—C11	175.8 (4)		
